# An Estimate of the Incidence of Prostate Cancer in Africa: A Systematic Review and Meta-Analysis

**DOI:** 10.1371/journal.pone.0153496

**Published:** 2016-04-13

**Authors:** Davies Adeloye, Rotimi Adedeji David, Adewale Victor Aderemi, Alexander Iseolorunkanmi, Ayo Oyedokun, Emeka E. J. Iweala, Nicholas Omoregbe, Charles K. Ayo

**Affiliations:** 1 Demography and Social Statistics, and the e-Health Research Cluster, Covenant University, PMB 1023, Ota, Ogun State, Nigeria; 2 Centre for Global Health Research and the World Health Organization Collaborating Centre for Population Health Research and Training, Usher Institute, University of Edinburgh, Edinburgh, United Kingdom; 3 Department of Surgery, Obafemi Awolowo University Teaching Hospitals Complex, Ile-Ife, Nigeria; 4 Department of Biochemistry, College of Health Sciences, Osun State University, Osogbo, Nigeria; 5 University Health Centre, Covenant University, Ota, Nigeria; 6 Saint Nicholas Hospital, Lagos, Nigeria; 7 Department of Biological Sciences, Covenant University, PMB 1023, Ota, Ogun State, Nigeria; 8 Department of Computer and Information Sciences, and the eHealth Research Cluster, Covenant University, PMB 1023, Ota, Ogun State, Nigeria; Carolina Urologic Research Center, UNITED STATES

## Abstract

**Background:**

Prostate cancer (PCa) is rated the second most common cancer and sixth leading cause of cancer deaths among men globally. Reports show that African men suffer disproportionately from PCa compared to men from other parts of the world. It is still quite difficult to accurately describe the burden of PCa in Africa due to poor cancer registration systems. We systematically reviewed the literature on prostate cancer in Africa and provided a continent-wide incidence rate of PCa based on available data in the region.

**Methods:**

A systematic literature search of Medline, EMBASE and Global Health from January 1980 to June 2015 was conducted, with additional search of Google Scholar, International Association of Cancer Registries (IACR), International Agency for Research on Cancer (IARC), and WHO African region websites, for studies that estimated incidence rate of PCa in any African location. Having assessed quality and consistency across selected studies, we extracted incidence rates of PCa and conducted a random effects meta-analysis.

**Results:**

Our search returned 9766 records, with 40 studies spreading across 16 African countries meeting our selection criteria. We estimated a pooled PCa incidence rate of 22.0 (95% CI: 19.93–23.97) per 100,000 population, and also reported a median incidence rate of 19.5 per 100,000 population. We observed an increasing trend in PCa incidence with advancing age, and over the main years covered.

**Conclusion:**

Effective cancer registration and extensive research are vital to appropriately quantifying PCa burden in Africa. We hope our findings may further assist at identifying relevant gaps, and contribute to improving knowledge, research, and interventions targeted at prostate cancer in Africa.

## Introduction

Cancer already constitutes a major public health burden globally [1]. Over the last 20 years, an increasing trend has been observed in the new cases and deaths from different cancers worldwide, especially in low-and middle-income countries (LMICs), owing to varying lifestyle and behavioural patterns, and geographic and environmental factors [2, 3]. In 2012 alone, there were 14.1 million new cases and 8.2 million deaths from cancer worldwide [4]. This burden is further expected to rise, with over 75 million prevalent cases, 27 million incident cases and 17 million cancer deaths expected globally by 2030 [5, 6]. Evidence suggests that most new cases of cancers are now found in Africa and LMICs, increasing from 15% in 1970, to 56% in 2008, and projected to reach about 70% by 2030 [1, 7, 8]. These are mostly related with rapid population growth, increasing life expectancy, urbanization with progressively westernized lifestyles, and high prevalence of HIV/AIDS in this region [9]. In 2012, Ferlay et al. reported that age specific incidence rates of cancer from all sites in Africa were 115.6 and 132.4 per 100,000 among men and women, respectively [4]. According to the 2011 United Nations high level committee on Non Communicable Diseases (NCDs), the focus of many stakeholders has turned towards addressing the growing burden of cancers and other non-communicable diseases (NCDs) [10], especially in Africa and other LMIC, where an existing high burden from infectious diseases has contributed to a prevalent double burden of disease [11].

Worldwide, prostate cancer (PCa) is rated the second most common cancer and sixth leading cause of cancer deaths among men, with over 1.1 million cases and 300,000 deaths estimated in 2012 [3, 4, 12]. The 2013 Institute for Health Metrics and Evaluation (IHME) study further reported an increasing disability adjusted life years (DALYs) and mortality from PCa, with an estimated 61% and 83% increase in DALYs and deaths from PCa respectively between 1990 and 2013 [13, 14]. In sub-Saharan Africa (SSA) alone, IHME estimated that DALYs from prostate cancer increased from 100,200 in 1990 to 219,700 in 2010, and deaths also increased from 5,600 to 12,300 over the same period [15, 16]. In the GLOBOCAN 2012 reports PCa incidence and mortality rates in Africa were reported to be 23.2 and 17.0 per 100,000, respectively [4]. While this was relatively lower than reported in some other world regions, some authors argued that African men suffer disproportionately from PCa compared to many parts of the world [17]. In fact, evidence shows that mortality rates from PCa are generally higher in predominantly black African populations compared to other races [18]. These variations were also observed in the patterns and presentations of PCa between northern and sub-Saharan African regions [19, 20]. Reports show that lower PCa incidence and mortality rates were observed in northern Africa at 10.6 and 7.0 per 100,000, compared to the average rates in SSA with 34.3 and 22.1 per 100,000, respectively [4]. Some of these variations have been attributed to the relatively higher poverty levels, dietary differences, genetic differences and the presence of infectious diseases in SSA [7]. Moreover, some challenges involved in the management of PCa have also been partly responsible. These include absence of low-cost, community-based screening and health promotion programmes, late presentation of patients to health facilities (usually at advanced stages of the malignancy), fewer options of treatment, high cost and/or unavailability of appropriate medications, lack of adequate follow-up, and inherent social norms and beliefs [12, 21].

Despite this seemingly disproportionate burden and the prevailing challenges, it is still quite difficult to precisely describe the burden of PCa in Africa due to weak health management information systems [3, 17, 22]. Many studies reported that poor routine record-keeping in most health facilities in Africa has been responsible for a relative lack of information and data on cancers and many health issues on the continent, with this greatly affecting research output, public health interventions and policy response [23–25]. Only few countries in Africa have population-based cancer registries, and these are not well-equipped and regularly updated [12, 26]. Ferlay and colleagues, for instance, reported that due to the lack of data on cancers observed across many countries especially in Africa, some of the country estimates provided in their report were corrected for under-reporting or incompleteness of cancer registration [4]. Odedina et al. also noted that due to unreliability of cancer registries from Africa and many developing countries, data from these settings may have been excluded in the estimation of global cancer burden by some authors; extrapolation were mainly based on data derived from a contextually similar country with relatively better registration systems [17].

We therefore aimed to systematically review the literature on prostate cancer in Africa and provide a continent-wide incidence rate based on available data in the region. We hope our findings may further assist at identifying relevant gaps, and also contribute to improving knowledge, research, and public health and policy interventions targeted at prostate cancer in Africa.

## Methods

### Search strategy and data sources

Medical Subject Headings (MESH) and keywords were identified and a final search strategy was developed. A systematic search of Medline, EMBASE and Global Health was conducted, with publication dates set from 1980 up to June 2015. As most cancer studies were registry-based and these may be unpublished, we conducted additional searches of Google Scholar, International Association of Cancer Registries (IACR), International Agency for Research on Cancer (IARC), and WHO African region websites to gather more data. Global publications on cancer were further reviewed; these include: the “GLOBOCAN studies”, “Cancer Incidence in Five Continents (CI5) series” and “Cancer in Africa: Epidemiology and Prevention”. For studies that were identified in the databases’ searches, any additional data (when available) on these studies were crosschecked and matched with our dataset. For identified key authors and relevant search terms, an e-mail alert was registered. Hand-searching of reference lists of all relevant publications, and key journals, were finally done to identify more studies that could have been omitted from the databases’ search. The list of African countries included in the search was based on the World Bank list of economies [27]. The search terms employed are shown in Table 1.

**Table 1:** Search terms for studies of prostate cancer in Africa.

*#*	*Searches*
**1**	africa/ or africa, northern/ or algeria/ or egypt/ or libya/ or morocco/ or africa, central/ or cameroon/ or central african republic/ or chad/ or congo/ or "democratic republic of the congo"/ or equatorial guinea/ or gabon/ or africa, eastern/ or burundi/ or djibouti/ or eritrea/ or ethiopia/ or kenya/ or rwanda/ or somalia/ or sudan/ or tanzania/ or uganda/ or africa, southern/ or angola/ or botswana/ or lesotho/ or malawi/ or mozambique/ or namibia/ or south africa/ or swaziland/ or zambia/ or zimbabwe/ or africa, western/ or benin/ or burkina faso/ or cape verde/ or cote d'ivoire/ or gambia/ or ghana/ or guinea/ or guinea-bissau/ or liberia/ or mali/ or mauritania/ or niger/ or nigeria/ or senegal/ or sierra leone/ or togo/
**2**	exp vital statistics/ or exp incidence/
**3**	(incidence* or prevalence* or morbidity or mortality).tw.
**4**	(disease adj3 burden).tw.
**5**	exp "cost of illness"/
**6**	exp quality-adjusted life years/
**7**	QALY.tw.
**8**	Disability adjusted life years.mp.
**9**	(initial adj2 burden).tw.
**10**	exp risk factors/
**11**	2 or 3 or 4 or 5 or 6 or 7 or 8 or 9 or 10
**12**	exp prostate cancer/
**13**	1 and 11 and 12

### Study selection

We included population-, hospital, and/or registry-based studies on prostate cancer conducted primarily on African population groups, and providing numerical estimates on the incidence of prostate cancer. We excluded studies that had non-human subjects, those conducted before 1980, and others that were mainly reviews, viewpoints or editorials. No language restrictions were applied.

### Case definitions

From an initial scoping exercise, it was evident that there were not many original population based cross-sectional or cohort studies on prostate cancers in Africa. Most of the relevant studies were mainly population-based or hospital-based registries’ surveys. We identified that the difficulties in cancer diagnosis and lack of trained health staffs (including oncologists) in many parts of Africa have limited ongoing research efforts. In our preliminary search, we looked for studies that identified PCa based on clinical suspicion, suggestive prostate specific antigen (PSA) assay and histological diagnosis. These were not always provided due to incompleteness of many cancer registries in Africa. This understanding ultimately shaped the overall conduct of this systematic review.

Based on the above, the case definitions needed to comply with any or a combination of these three:

1. Studies reporting laboratory-confirmed cases evident from elevated PSA and histology;
2. Studies reporting diagnosis of prostate cancer confirmed by a recognized medical practitioner; and/or
3. Studies with incident cases of cancers classified according to the primary anatomic site (topography) and cellular characteristics (morphology including histology, behaviour, and grade) in accordance with ICD-O and ICD guidelines [28–30]

### Quality criteria

To ensure a relatively high quality of studies, we further assessed studies for flaws in the design and collation of data, and if the limitations were explicitly stated. As studies were mainly registry-based, we checked for active or passive cancer registration process, and cancer diagnosis, and/or morphologic verification. We assessed the appropriateness of statistical and analytical methods in the estimation of cancer incidence. As we intended to pool a continent-wide cancer-estimate from all studies, we also assessed studies for heterogeneities within and outside various population groups and if each study was representative of a larger population in the region that can be generalized to the overall African population.

### Data extraction and analysis

All extracted data were stored in 2010 Microsoft Excel file format. Data was extracted systematically on study location, study period, mean age or age range, population size, and incident cases. Some studies only reported number of PCa cases without any incidence rate and/or reference population denominator from which incidence rate can be calculated. We therefore applied the United Nations demographic projections for the country (for the study period) to determine the incidence rate. Data were then sorted into five main African regions (central, east, north, south, and west) and countries represented within each region. From extracted incidence rates of prostate cancer, we conducted a random effects meta-analysis [31], with pooled incidence rate expressed per 100,000 population. Overall pooled incidence rates for Africa and each country represented were estimated. All statistical analyses were conducted on Microsoft Excel and Stata 13.1 (Copyright 1985–2013 Stata Corp LP). The PRISMA checklist [32] was employed, and guided the overall conduct of this study (S1 Checklist).

## Results

### Search results

**Fig 1:**
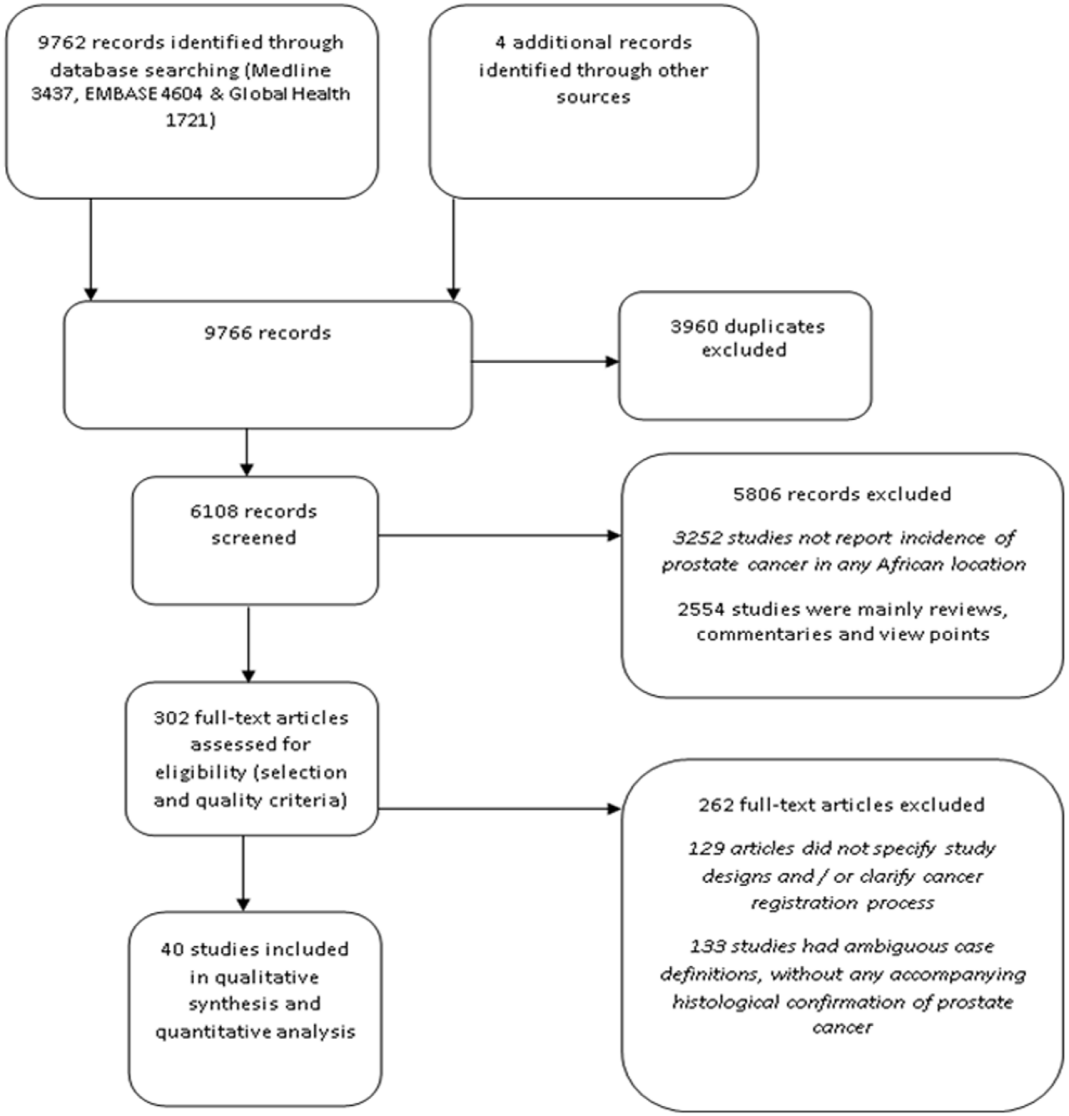
Flow chart of search results.

The literature search returned a total of 9766 publications. Out of these, 9762 publications were from three databases- Medline (3437), EMBASE (4604) and Global Health (1721). The remaining four studies were selected from other sources earlier described in the methods. After all studies have been collated and duplicates removed, 6108 records remained. On screening titles for relevance (Studies providing incidence of PCA in any African location), 5806 studies were excluded, giving a total of 302 full texts that were assessed. After applying the quality criteria, 262 studies were excluded (129 articles did not specify study designs and / or clarify cancer registration process, and 133 studies had ambiguous case definitions, without any accompanying histological confirmation of prostate cancer). A total of 40 studies were finally retained for the review (Fig 1).

### Study characteristics

The retained 40 studies [33–72] were conducted across 16 African countries with Nigeria having the highest records (17 studies) (Table 2). Across African regions, Central Africa had 3 studies, Eastern Africa 2 studies, Northern Africa 4 studies, Southern Africa had 9 studies, and Western Africa 22 studies. 66 data points were extracted in all with Nigeria and South Africa having highest points at 24 and 22, respectively (S1 Table).

**Table 2:** Study characteristics.

Country, Location/Registry	African region	Study period	Study population	Age	Diagnostic criteria	Confirmation of diagnosis (%)	Incidence (per 100000)
Cameroon, Yaounde [33]	Central	1986–1990	41,578	67.7	ICD	Histologically verified	93.8
Cameroon, Yaounde [34]	Central	2004–2011	1,299,369	44.8	CanReg5	Morphologically verified	11.6
Rwanda, Butare [35]	Central	1991–1994	147,001	46.5	ICD-9	-	1.02
Uganda, Kampala [36]	East	1991–2006	1,674,384	44.5	ICD-O-2	-	35.5
Uganda, Kyadondo [37]	East	1995–1997	577,200	43.2	ICD-O (Percy et al. 1990); ICD-10 for tabulation	Morphologically verified	39.2*
Egypt, Menofeia [38]	North	1992–1996	2,755,000	53.5	Diagnosed by tumor site according to the ICD-9	-	0.41
Libya, Benghazi [39]	North	2003	660,147	53	ICD-O-3	Microscopically / histologically verified	11.4
Libya, Eastern region [40]	North	2004	372,196	51.5	ICD-10		8.3
Tunisia, Souse [41]	North	1993–2006	2,760,900	50.1	ICD-10	Morphologically verified	11.9
Malawi, Blantyre [42]	South	1994–98	782,000	48.5	Histopathology, ultrasound and endoscopy, radiography, biochemical tests (AFP). ICD-O-2 used for data entry, but converted to ICD-10 for tabulation and morphology	Morphological (histological/cytological) verification	5.5
South Africa [43]	South	1986–2006	15,035,714	67.8	ICD-10, ICD-O-3	Histologically verified	30.8*
South Africa, Eastern Cape [44]	South	1998–2002	1,292,959	47.7	ICD-10, ICD-O	Histologically verified	4.4
South Africa, Eastern Cape [45]	South	2002–2012	**724,520**	66.9	ICD-10, ICD-O-3	Histologically verified	9.90*
South Africa, Johannesburgh [46]	South	1998–1999	843,102	54.8	ICD-9, ICD-O	Active case finding	17.2
Swaziland [47]	South	1996–1999	231,102	55.5	ICD-O		21.5
Zambia, Lusaka [48]	South	1990–2005	1,084,703	67	ICD	Histologically verified	37.7
Zimbabwe, Harare [49]	South	1990–1992	537,244	51.5	ICD-O, ICD-10	Death certificates only (DCO)	29.2
Zimbabwe, Harare [50]	South	1993–1995	650,600	48.2	ICD-O, converted to ICD-10 for analysis	Histologically verified	26
Cote d'Ivoire, Abidjan [51]	West	1995–1997	1,000,342	38.8	Histopathology- coded using ICD-0 2nd edition, converted to ICD-10 for tabulation	Morphologic verification	31.4
Gambia [52]	West	1988–1997	1,340,000	49.5	ICD-0, ICD 10	Histologically verified	2.5
Gambia [53]	West	1998–2006	427,176	49.7	ICD-0-3, ICD 10		3.46
Guinea, Conakry [54]	West	1992–1995	6,700,000	51.1	Tumor site and histology have been coded using the ICD-0 first edition (WHO, 1976). These codes were converted to ICD-9 for tabulation	Morphologically verified	8.1
Mali, Bamako [55]	West	1987–1988	646,163	52.1	ICD-O (WHO 1976), ICD-9 (Percy & Van Holten, 1979)	Death certificate only	4.7
Nigeria [56]	West	**1984–2004**	21,014,655	65.3	ICD	Histologically verified	16.42, 16.31* (SS), 4.38 (SW), 0.88 (NC), 5.28 (NW)
Nigeria [57]	West	1990–1996	1,173,422	67.5	ICD-9	-	3.88*
Nigeria [58]	West	1994	111,000	68.3	ICD	Histologically verified	127.0
Nigeria, Abuja & Ibadan [59]	West	2009–2010	3,175,413	49.1	ICD-O-3	-	25.9 (Abuja), 17.4 (Ibadan)
Nigeria, Calabar [60]	West	2002	236,542	66.6	ICD-O	Histologically verified	61.3
Nigeria, Ile-Ife [61]	West	2002–2004	103,562	68	ICD-9	Histologically verified	182.5
Nigeria, Ilorin [62]	West	1989–1998	847,582	69	ICD	Histologically verified	58.2
Nigeria, Kano [63]	West	1998–2002	9,383,682	63.7	ICD	Histologically verified	0.72
Nigeria, Lagos [64]	West	**2005–2011**	11,200,000	66	ICD-10, ICD-O-3	Histologically verified	0.375
Nigeria, Lagos [65]	West	2012	11,200,000	60.8	ICD-10, ICD-O-3	Histologically verified	0.38
Nigeria, Maiduguri [66]	West	1987–2004	1,197,497	71.2	ICD	Histologically verified	13.8
Nigeria, Nnewi [67]	West	2000	391,227	68.3	ICD-9	Histologically verified	4.1*
Nigeria, Port Harcourt [68]	West	1985–1998	154,594	71	ICD	Morphologically verified	114
Nigeria, Port Harcourt [69]	West	1997–2006	1,382,592	70	ICD-O	Histologically verified	14.3
Nigeria, Uyo [70]	West	2002–2012	139,073	64	ICD-9, ICD-O-3	Histologically verified	151
Nigeria, Zaria [71]	West	1991–2000	760,084	60	ICD-O	Histologically verified	19.9
Nigeria, Zaria [72]	West	1992–1996	408,198	69.8	ICD	Histologically verified	19.8

CD: International Classification of Diseases, ICD-O: International Classification of Diseases for Oncology,

*Estimate in the final year of study.

Less than 50% of studies were conducted in population-based cancer registries, which do not necessarily cover an entire country population; some only covered provinces, districts or major cities within a country. Most studies were conducted between 1990 and 2009, generating over 80% of the data points. Periods of studies ranged from 1 year to 18 years with a median study period of 5 years. Mean age across studies ranged from 38.8 years to 71.2 years, with subjects mostly in the 60–69 years age group (62%), followed by the 50–59 years age group (15%). Most studies reported a laboratory confirmed histological diagnosis of PCa (Table 2 & S1 Table).

### Pooled incidence rate of prostate cancer in Africa

From the 66 data point spreading across 16 African countries, the lowest PCa incidence rates were from two studies conducted in Lagos, southwest Nigeria between 2005 and 2011, with both reporting at 0.38/10,0000 population [64, 65]. The highest PCa incidence was also estimated in Ile-Ife, south west Nigeria, at 182.5/100,000 population [61].

**Fig 2:**
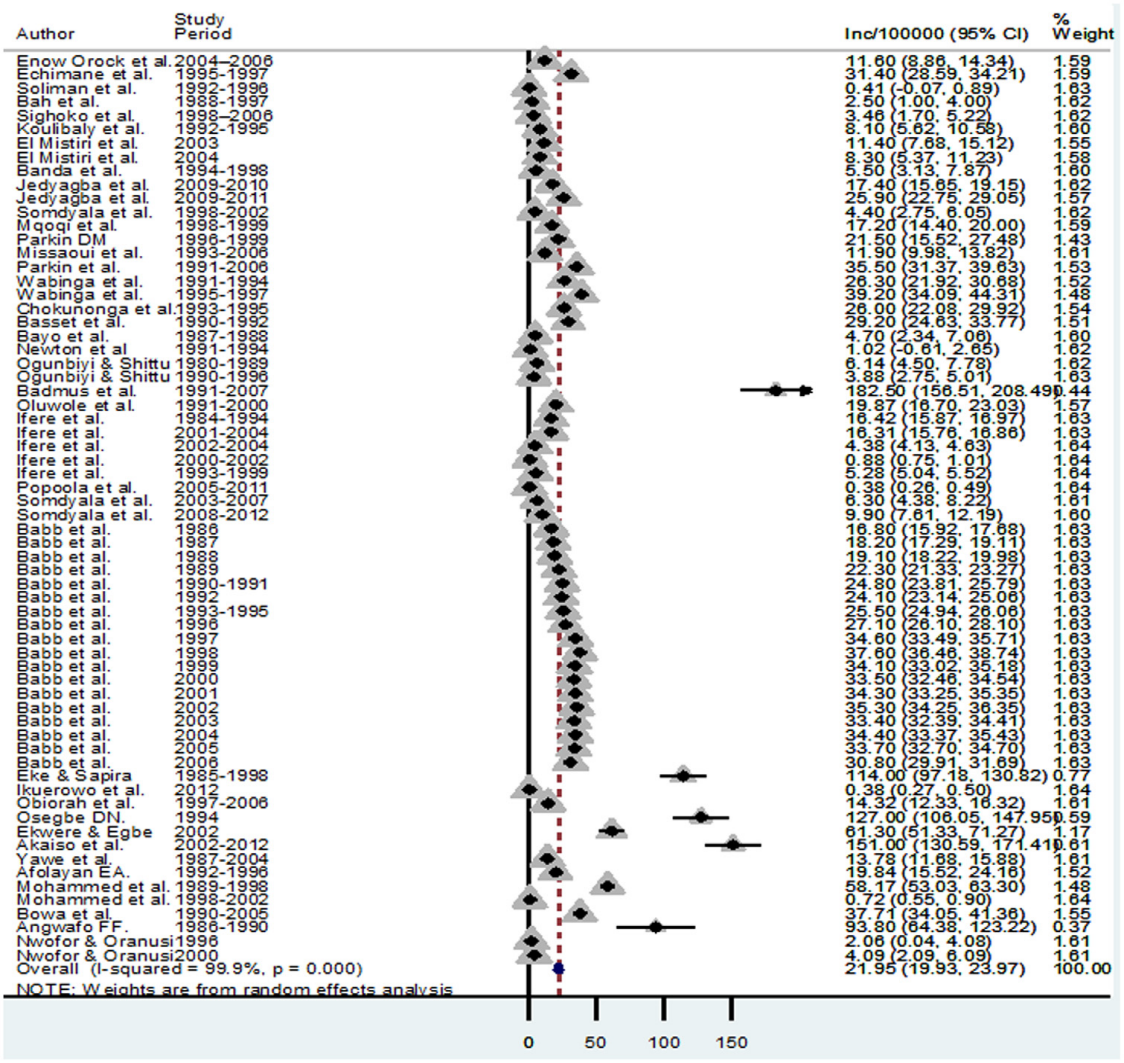
Forest plot showing pooled incidence of prostate cancer in Africa.

**Fig 3:**
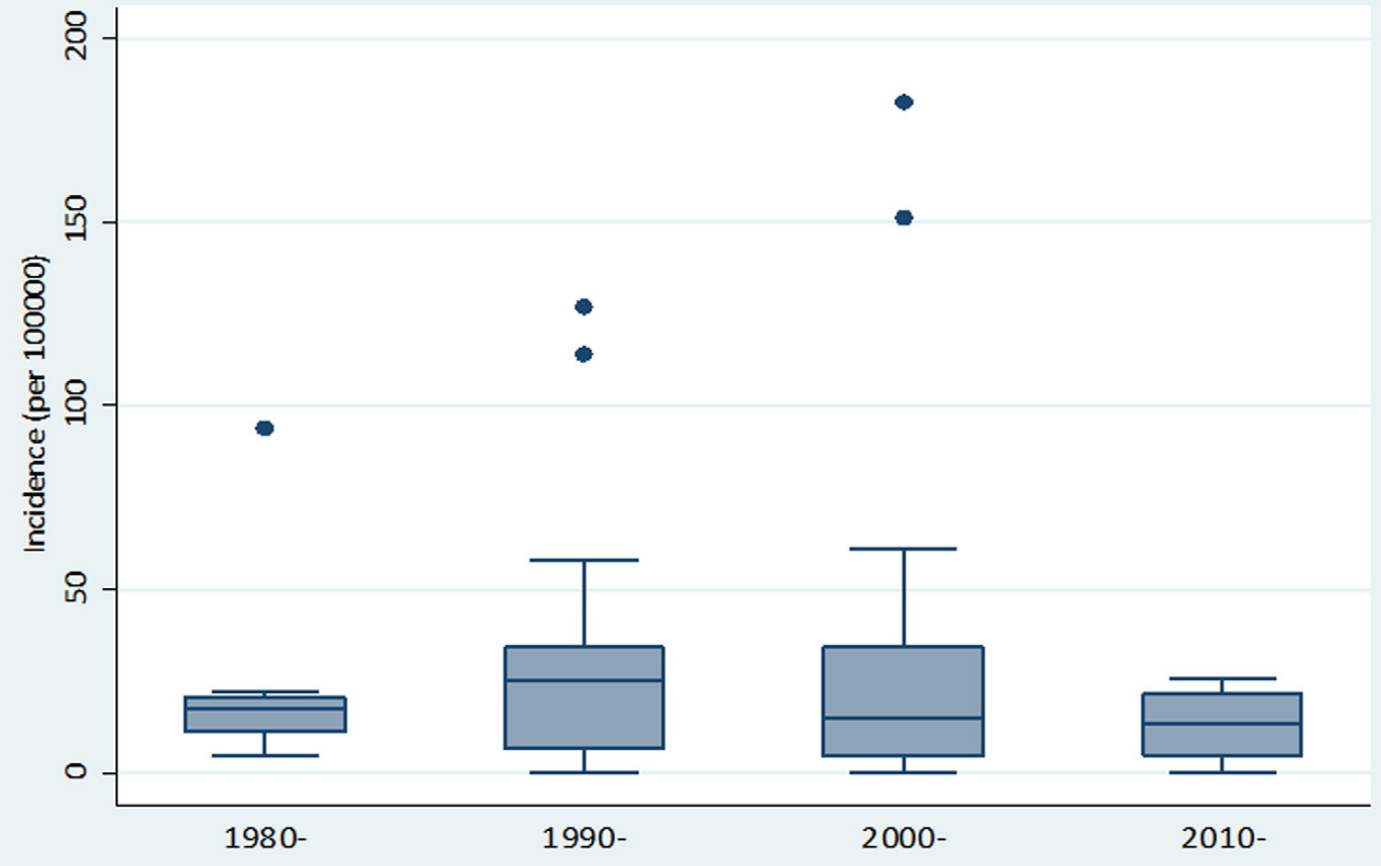
Distribution of PCa incidence rates over study periods.

**Fig 4:**
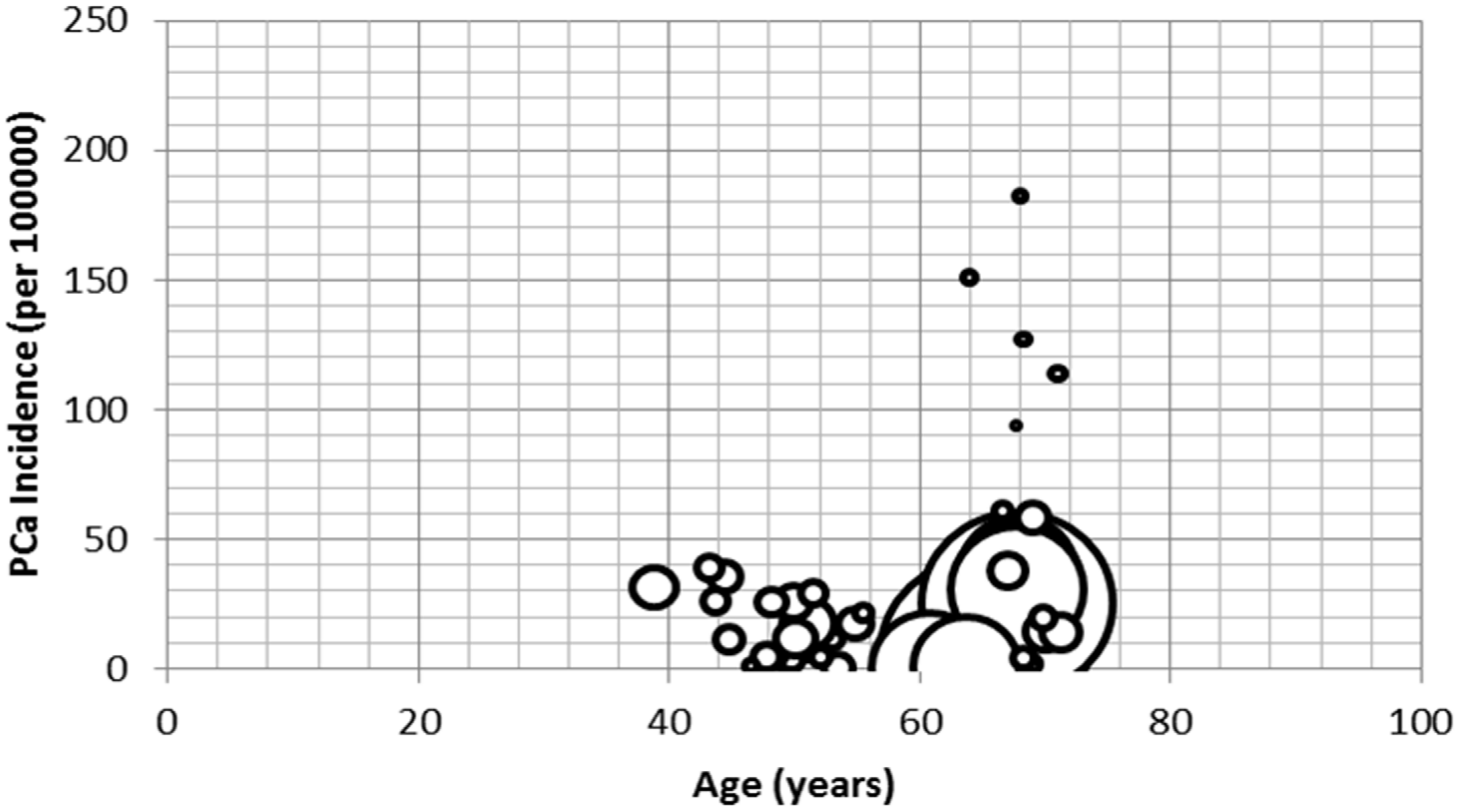
Distribution of PCa incidence rates over mean age (size of bubble corresponds to population size).

The overall pooled incidence of PCa in Africa was 21.95 (95% Confidence Interval (CI): 19.93–23.97)/100,000 population, with a median incidence of 19.47/100,000 population (Fig 2). Highest PCa incidence rates were estimated between 2000–2009 and 1990–1999 at 26.1 (95%CI: 21.0–31.2) and 21.9 (95% CI: 18.9–25.0) per 100,000 population, respectively. Between 1980–1989 and 2010–2015, PCa incidence rates were estimated at 15.7 (95%CI: 12.7–18.8) and 13.3 (1.6–25.1) per 100,000 population, respectively (Table 3 and Fig 3). In the age group analysis, the highest PCa incidence was 39.0 (95% CI: 19.9–53.3)/100,000 population estimated in people aged 70 years and above. This is followed by 60–69 years with a PCa incidence rate of 25.0 (95% CI: 22.4–27.5)/100,000 population. Incidence rates of 12.9 (95% CI: 7.0–18.7) and 16.3 (10.1–22.4) per 100,000 population were estimated in 40–49 years and 50–59 years, respectively (Table 4 and Fig 4). Applying this to the United Nations population estimates for Africa (assuming demographic factors and other population health determinants were fully accounted for), the pooled PCa incidence rate would amount to about 25,000 cases of PCa among men aged 40 years and above in Africa in 2015.

**Table 3:** Pooled PCa incidence rates over study periods.

Year	Data points	PCa incidence (per 100000)	95% CI (per 100000)
**1980–1989**	8	15.7	12.7–18.8
**1990–1999**	28	21.9	18.9–25.0
**2000–2009**	26	26.1	21.0–31.2
**2010–2015**	4	13.3	1.6–25.1
**Total**	66	22.0	20.0–24.0

**Table 4:** Pooled PCa incidence rates over age groups.

Age (years)	Data points	PCa incidence (per 100000)	95% CI (per 100000)
**40–49**	10	12.9	7.0–18.7
**50–59**	11	16.3	10.1–22.4
**60–69**	41	25.0	22.4–27.5
**70+**	3	39.0	19.9–53.3
**Total**	66	22.0	20.0–24.0

## Discussion

This study attempted to provide continent-wide pooled estimates of prostate cancer in Africa. Data employed were from population- and hospital-based cancer registries, with the pooled estimates provided based on a total population ranging from 42,000 to 15 million. There is relatively low level of research on PCa incidence in Africa, and/or across most African regions. While the GLOBOCAN studies have provided estimates on major cancer types in Africa over the years, there are still concerns if their estimates truly reflects the burden of cancer in the African population, especially due to non-availability of data across many parts of Africa [19, 73]. Ferlay et al. reported that in the GLOBOCAN 2012 study, country estimates were derived from datasets which have been corrected and modelled to account for under-reporting and incompleteness of cancer registries in each country [4]. This study therefore provides direct comparisons with the reported estimates. We assume the low incidence recorded in the Lagos studies may be connected with the relatively large population size in the metropolis, where many new PCa cases may not be picked by the cancer registry over the study period [64, 65]. While the higher incidence rate recorded in Ile-Ife may not necessarily be due to a more effective cancer registry, but could partly be reflecting a higher aging population with PCa (mean age 68) who present to hospitals in anticipation of better care and cure of the cancer [61]. From our study, we estimated a pooled PCa incidence rate of 22.0 (95% CI: 19.93–23.97)/100,000 population, and to allow detailed understanding of the overall data distribution, we also reported a median incidence rate of 19.5/100,000 population. Our figures are almost as reported by Ferlay et al., with an estimated incidence rate of 23.2/100,000 in 2012 [4]. This possibly validates our findings, and may suggest that Africa is still in this PCa incidence range, at least based on available data in the continent. We however think active cancer registration and extensive research are vital to appropriately quantifying this burden in Africa. Meanwhile, we also observed an increasing trend in PCa incidence with advancing age and over the years covered in the study (i.e. 1980–2009, excluding 2010–2015 where we retrieved only 4 data points, see Tables 3 & 4). This is also in line with many reports; for example, advancing age has been described as a major risk factor for prostate cancer, with a peak incidence among men aged 65 years or higher across many world regions [56]. Moreover, in 1990, incidence rates of prostate cancer were between 2.6–16.9/100,000 across many parts of Africa [20, 74], with this increasing to 4.7–38.1/100,000 in 2007 [75]. GLOBOCAN also estimated an incidence rate of 16.0/100,000 in 2002, with this increasing to 17.5/100,000 in 2008 [6, 76].

Generally, this study attempted to provide pooled incidence rate of prostate cancer in Africa, compare this with reported global estimates, identify gaps, and inform stakeholders on the problems arising from lack of detailed cancer registration in Africa. To the best of our knowledge, this review provides the first systematically derived pooled estimates of PCa incidence in Africa. However, this study could have been constrained by a number of factors. While the retained studies spread across various parts of Africa, only 16 African countries were represented and less than 50% of data were from population-based registries. Most of these cancer registries are restricted to specific locations in the respective countries, and do not all cover national populations. It is therefore difficult to say that the registry reports were representative of the total population of the country where the registry is based. In addition, this study mainly included very few registries from rural areas where more people generally reside in Africa and they typically reported lower cancer incidence rates, compared to the urban registries that reported higher incidence rates; this may thus potentially affect our estimates with an under-estimation of cancer burden in rural areas and an over-estimation in urban areas. Moreover, not all studies reported basis of cancer diagnosis, it was therefore based on evidences of epidemiological rigours and clinical case ascertainment (active/ passive case finding) that some studies were included. Another limitation is the widespread variations within and between study populations. For a combination of studies with such high heterogeneity, it may also not be appropriate to pool a summary effect. However, as reported earlier, there is very low research output in Africa, and the few studies have not been conducted under strict international guidelines, so heterogeneities can be expected. In addition, restricting our analysis to studies that have the same study design, same sampling frame and same case definition is almost impossible. Lastly, we still cannot say with all certainty how closely representative these estimates were of the larger African population, we however provided median PCa incidence rate to give an insight into the raw data distribution. However, this study provides ample evidence on the need for more detailed cancer data collation and registration across many African settings towards ensuring that a true burden of cancer is reflected.

### Challenges in the response to prostate cancer in Africa

The absence of effective low-cost PCa screening facilities and health promotion programmes across many African countries has been a major challenge in the management of PCa [77]. With the discovery of Prostate Specific Antigen (PSA) tumor marker, PCa has been reportedly diagnosed early in many asymptomatic patients on routine medical examination or screening [78]. There are however varying controversies among experts on the usefulness or otherwise of PSA screening for early prostate cancers [79]. For example, some investigators discovered that annual PSA screening may result in commencing treatment for slow-growing prostate cancers, or benign prostatic diseases, that are unlikely to threaten a man’s life, thereby exposing the men needlessly to the potential adverse effects of over-treatment [80]. Nonetheless, PSA, digital rectal examination and detailed medical history are still the preferred options in many settings in conjunction with appropriate health education and promotion programmes [79]. Large scale population-based prostate screening is not yet common in Africa, as resources are far too limited to be diverted to cancer diagnosis in the face of other health challenges [65, 75, 81]. Many prostate cancer cases therefore present late with symptoms of local invasion or distant metastasis [78].

According to Parkin et al., incidence data on cancers are better sourced jointly from hospital-based and population-based cancer registries, as this will allow new cancer cases to be sourced from within and outside the hospital [23]. According to the WHO, many African countries still do not have a functional cancer registration system [7]. The few cancer registries in these regions only cover the urban cities and do not really spread to the rural areas [82]. In fact, only 5 cancer registries from 5 African countries contributed to the first volume of “Cancer Incidence in Five Continents” [83, 84]. It was estimated that in 1990, only 5% of African countries were covered by functional cancer registries, and this increased to about 20% in 2008 [6]. The WHO further reported that cancer registration globally has progressed haphazardly over the last two decades as most countries do not have an official policy to support cancer registration [7]. Moreover, resource allocation for cancer registration is low in many developing countries as this is perceived a luxury amidst other challenging health issues [84]. Vital registration systems are also important sources of mortality data on cancers [85]. Despite about 42% global vital registration coverage as at 1990, the current coverage in Africa has been reported to be incomplete and unimaginably low [85]. The lack of data, coupled with availability of funds, has further resulted in the dearth of basic and clinical research on PCa in Africa [9]. There is limited number of clinical trials to test new treatments, and lack of support for basic research to discover new therapeutic agents and predictive molecular diagnostics [17]. There are few valid and methodologically comprehensive epidemiological studies to systematically correct the profound lapses in population-based data on PCa incidence rates in Africa [9].

The treatment and overall management of PCa has been constrained by late presentation, advanced disease, and scarcity of urologists, pathologists, radiotherapy and androgen-deprivation therapies [86]. Bilateral total orchidectomy has been the preferred treatment option in many African settings because it is relatively cheap and simple to perform, but this is still not without its challenges [61]. In many African settings, urologists also lack the expertise to effectively perform curative radical prostatectomies, and this is further complicated by the shortage of artificial sphincters and relevant devices useful in management of possible complications from the procedure [87, 88]. Radiotherapy has an important role in the management of PCa in Africa [89]. However, oncologists have reported that the use of radiotherapy is limited in Africa owing to lack of adequate medical infrastructure, specialty centres, radiotherapists and technical expertise in many African regions [90]. In 2013, research findings showed that only 23 countries in Africa offer radiotherapy, with external beam radiotherapy being the most widely used [89]. Additionally, the use of chemotherapy in Africa is often limited by cost and non-availability of the drugs [19]. Relatively newer therapies such as cryotherapy, high intensity focused ultrasound, androgen biosynthesis inhibitors and therapeutic vaccines are yet to be fully established in Africa [19, 61]. Even in many developed economies where some of these newer approaches are in use, there are reports that the responses are only temporary as patients develop resistance resulting such as the metastatic castration-resistant prostate cancer [91, 92], underscoring a need for yet more targeted therapies [93, 94]. Meanwhile, some palliative care initiatives have been created in some parts of Africa. The African Palliative Care Association (APCA) works with many African health care providers to relieve pains and improve overall wellbeing of people at advanced stages of cancers and other terminal illnesses [95, 96]. However, reports have shown that these initiatives have been limited by the unavailability and unaffordability of effective pain-relieving medications in many settings [95]. According to a 2004 survey, only five African countries have cancer palliative care programmes and/ or allow the dispensing of oral morphine [96, 97], with this only increasing to 28 out 57 African countries in 2010 [98]. It was further observed that with strong political will, increased funding, capacity building and establishment of improved treatment facilities, sustainable palliative care programmes may be established in Africa [98], as this was the case in Uganda, which is presumably the only country in the world where nurses are allowed to prescribe morphine [99].

## Conclusion

Prostate cancer contributes significantly to the public health burden in Africa. Yet, due to data gaps, the exact burden is still far from known. With continued urbanization, population growth and increasing life expectancies in Africa, its burden is still expected to increase. The response from the government of many African nations still remains a huge concern. There is need for urgent re-prioritization of health programmes in Africa towards improving research and training, screening, diagnosis, treatment, cancer registration, data handling and the overall management of prostate cancer in the region.

## Supporting Information

S1 Checklist PRISMA Checklist. (DOC)

S1 Table Dataset extracted from all studies. This table shows complete dataset extracted from all studies retained in the review.
(DOC)
