# Supplementary material for: An Estimate of the Incidence of Prostate Cancer in Africa: A Systematic Review and Meta-Analysis
Source: PLoS One. 2016 Apr 13;11(4):e0153496. doi: 10.1371/journal.pone.0153496 (PMC4830589; doi:10.1371/journal.pone.0153496)
Supplement: S1 Table — This table shows complete dataset extracted from all studies retained in the review. (DOC) [file pone.0153496.s002.doc]

# S1 Table. Dataset extracted from all studies

| **Author** | **Country** | **Study period** | **Mean age** | **Cases** | **Population** | **Incidence/ 100000** |
| --- | --- | --- | --- | --- | --- | --- |
| Enow Orock et al. | Cameroon | 2004–2006 | 44.8 | 342 | 592112 | 11.55 |
| Echimane et al. | Cote d'Ivoire | 1995-1997 | 38.8 | 322 | 1531177 | 31.40 |
| Soliman et al. | Egypt | 1992-1996 | 53.5 | 14 | 689921 | 0.41 |
| Bah et al. | Gambia | 1988-1997 | 49.5 | 51 | 427176 | 2.50 |
| Sighoko et al. | Gambia | 1998–2006 | 49.7 | 95 | 427176 | 3.46 |
| Koulibaly et al. | Guinea | 1992-1995 | 51.1 | 39 | 507469 | 8.10 |
| El Mistiri et al. | Libya | 2003 | 53 | 45 | 316870 | 11.40 |
| El Mistiri et al. | Libya | 2004 | 51.5 | 44 | 372196 | 8.30 |
| Banda et al. | Malawi | 1994-1998 | 48.5 | 45 | 375364 | 5.50 |
| Jedyagba et al. | Nigeria | 2009-2010 | 51.1 | 251 | 2175412 | 17.40 |
| Jedyagba et al. | Nigeria | 2009-2011 | 49.9 | 107 | 1000001 | 25.90 |
| Somdyala et al. | South Africa, East Cape | 1998-2002 | 47.7 | 69 | 620620 | 4.40 |
| Mqoqi et al. | South Africa | 1998-1999 | 54.8 | 197 | 843102 | 17.20 |
| Parkin DM | Swaziland | 1996-1999 | 55.5 | 132 | 231102 | 21.50 |
| Missaoui et al. | Tunisia | 1993-2006 | 50.1 | 319 | 1242405 | 11.90 |
| Parkin et al. | Uganda | 1991-2006 | 44.5 | 1639 | 800990 | 35.50 |
| Wabinga et al. | Uganda | 1991-1994 | 43.7 | 113 | 526900 | 26.30 |
| Wabinga et al. | Uganda | 1995-1997 | 43.2 | 139 | 577200 | 39.20 |
| Chokunonga et al. | Zimbabwe | 1993-1995 | 48.2 | 134 | 650600 | 26.00 |
| Basset et al. | Zimbabwe | 1990-1992 | 51.5 | 112 | 537244 | 29.20 |
| Bayo et al. | Mali | 1987-1988 | 52.1 | 12 | 323383 | 4.70 |
| Newton et al | Rwanda | 1991-1994 | 46.5 | 6 | 147001 | 1.02 |
| Ogunbiyi & Shittu | Nigeria | 1980-1989 | 67.5 | 540 | 878921 | 6.14 |
| Ogunbiyi & Shittu | Nigeria | 1990-1996 | 67.5 | 319 | 1173422 | 3.88 |
| Badmus et al. | Nigeria | 2002-2004 | 68 | 189 | 103562 | 182.50 |
| Oluwole et al. | Nigeria, Zaria | 1991-2000 | 60 | 151 | 760084 | 19.87 |
| Ifere et al. | Nigeria, Southsouth | 1984-1994 | 65 | 3451 | 21014655 | 16.42 |
| Ifere et al. | Nigeria, Southsouth | 2001-2004 | 65 | 3427 | 21014655 | 16.31 |
| Ifere et al. | Nigeria, Southwest | 2002-2004 | 65 | 1208 | 27581992 | 4.38 |
| Ifere et al. | Nigeria, Northcentral | 2000-2002 | 65 | 178 | 20266257 | 0.88 |
| Ifere et al. | Nigeria, Northwest | 1993-1999 | 65 | 1890 | 35786998 | 5.28 |
| Popoola et al. | Nigeria, Lagos | 2005-2011 | 66 | 42 | 11200000 | 0.38 |
| Somdyala et al. | South Africa, East Cape | 2003-2007 | 67.8 | 41 | 654682 | 6.30 |
| Somdyala et al. | South Africa, East Cape | 2008-2012 | 66.9 | 72 | 724520 | 9.90 |
| Babb et al. | South Africa | 1986 | 67.8 | 1401 | 8339286 | 16.80 |
| Babb et al. | South Africa | 1987 | 67.8 | 1522 | 8362637 | 18.20 |
| Babb et al. | South Africa | 1988 | 67.8 | 1800 | 9424084 | 19.10 |
| Babb et al. | South Africa | 1989 | 67.8 | 2046 | 9174888 | 22.30 |
| Babb et al. | South Africa | 1990-1991 | 67.8 | 2434 | 9814516 | 24.80 |
| Babb et al. | South Africa | 1992 | 67.8 | 2424 | 10058091 | 24.10 |
| Babb et al. | South Africa | 1993-1995 | 67.8 | 7862 | 30831373 | 25.50 |
| Babb et al. | South Africa | 1996 | 67.8 | 2802 | 10339483 | 27.10 |
| Babb et al. | South Africa | 1997 | 67.8 | 3715 | 10736994 | 34.60 |
| Babb et al. | South Africa | 1998 | 67.8 | 4171 | 11093085 | 37.60 |
| Babb et al. | South Africa | 1999 | 67.8 | 3860 | 11319648 | 34.10 |
| Babb et al. | South Africa | 2000 | 67.8 | 3958 | 11814925 | 33.50 |
| Babb et al. | South Africa | 2001 | 67.8 | 4118 | 12005831 | 34.30 |
| Babb et al. | South Africa | 2002 | 67.8 | 4318 | 12232295 | 35.30 |
| Babb et al. | South Africa | 2003 | 67.8 | 4178 | 12508982 | 33.40 |
| Babb et al. | South Africa | 2004 | 67.8 | 4301 | 12502907 | 34.40 |
| Babb et al. | South Africa | 2005 | 67.8 | 4346 | 12896142 | 33.70 |
| Babb et al. | South Africa | 2006 | 67.8 | 4631 | 15035714 | 30.80 |
| Eke & Sapira | Nigeria, Portharcourt | 1985-1998 | 71 | 47 | 154594 | 114.00 |
| Ikuerowo et al. | Nigeria, Lagos | 2012 | 60.8 | 43 | 11200000 | 0.38 |
| Obiorah et al. | Nigeria, Port Harcourt | 1997-2006 | 70 | 198 | 1382592 | 14.32 |
| Osegbe DN. | Nigeria | 1994 | 68.3 | 141 | 111000 | 127.00 |
| Ekwere & Egbe | Nigeria, Calabar | 2002 | 66.6 | 145 | 236542 | 61.30 |
| Akaiso et al. | Nigeria, Uyo | 2002-2012 | 64 | 210 | 139073 | 151.00 |
| Yawe et al. | Nigeria, Maiduguri | 1987-2004 | 71.2 | 165 | 1197497 | 13.78 |
| Afolayan EA. | Nigeria, Zaria | 1992-1996 | 69.8 | 81 | 408198 | 19.84 |
| Mohammed et al. | Nigeria, Ilorin | 1989-1998 | 69 | 493 | 847582 | 58.17 |
| Mohammed et al. | Nigeria, Kano | 1998-2002 | 63.7 | 68 | 9383682 | 0.72 |
| Bowa et al. | Zambia, Lusaka | 1990-2005 | 67 | 409 | 1084703 | 37.71 |
| Angwafo FF. | Cameroon, Yaounde | 1986-1990 | 67.7 | 39 | 41578 | 93.80 |
| Nwofor & Oranusi | Nigeria, Nnewi | 1996 | 68.9 | 4 | 193987 | 2.06 |
| Nwofor & Oranusi | Nigeria, Nnewi | 2000 | 68.3 | 16 | 391227 | 4.09 |
